# How to Stimulate Continuous Innovative Knowledge Contribution? Mediation by Self-Efficacy and Moderation by Knowledge Level

**DOI:** 10.3390/bs14080691

**Published:** 2024-08-09

**Authors:** Jun Wang, Shan Jiang, Ou Liu, Yani Wang

**Affiliations:** 1 School of Economics and Management, Beihang University, No. 37 Xueyuan Road, Haidian District, Beijing 100191, China; king.wang@buaa.edu.cn; 2 Key Laboratory of Complex System Analysis, Management and Decision, Beihang University, Ministry of Education, Beijing 100191, China; 3 School of Artificial Intelligence, Beihang University, No. 37 Xueyuan Road, Haidian District, Beijing 100191, China; 4 International Business School Suzhou, Xi’an Jiaotong-Liverpool University, Suzhou 215123, China; ou.liu@xjtlu.edu.cn; 5 Yanjing Medical College, Capital Medical University, Beijing 101300, China; wangyani@ccmu.edu.cn

**Keywords:** continuous innovative knowledge contribution, innovation, self-efficacy, knowledge level, community relations, participation value

## Abstract

Taking the perspective of innovative knowledge management, this study aimed to investigate the stimulation mechanism of continuous innovative knowledge contribution (CIKC). Through a quantitative study conducted in an open innovation community, we modeled a stimulus–organism–response framework to conduct a path analysis from the external environment to internal cognition, and then to knowledge contribution, and filled in the gaps in the mediating and moderating mechanisms. We focused on the stimuli of knowledge contribution, in view of both quantity and quality. Panel data from six periods in one year was collected for dynamic analysis, and we used the fixed effect model to test our hypotheses of mediation effect, moderation effect, and mediated-moderation effect. There were some interesting findings, showing that user’s self-efficacy plays a partial mediating role in the quantity, rather than the quality, of CIKC; meanwhile, the users’ knowledge level plays a moderating role, and there is a negative moderating mechanism of knowledge level in the process from participation value to the quality of CIKC.

## 1. Introduction

In the era of the knowledge economy, innovation has become an important driving force for social progress and economic development [1,2,3]. Innovative enterprises must become more competitive for their sustainable development [4,5] by continuous “open innovation”. This concept was first proposed by Chesbrough in 2003 and has become a new model of enterprise innovation [6] to break through organizational boundaries, integrate internal and external resources, stimulate innovation vitality, and enhance the openness of innovation [1,7,8].

In recent years, the virtual community has achieved great development, relying on the internet to build a society of information and data [9]. Open innovation community (OIC), as a kind of virtual community, has become an important outcome of modern IT [10]. In order to improve innovation capabilities, many enterprises have developed virtual innovation platforms to improve the utilizing efficiency of diverse knowledge, and to make full use of the advantages of internal and external innovation resources [7,11]. OICs can help pluralize and ‘open up’ possible innovation trajectories [12]. OICs are currently increasingly being embraced by many companies, bring together user groups interested in innovation activities, and obtain and augment various forms of innovation resources, such as a wealth of innovative ideas, design solutions, and product, or service feedback, through the knowledge content contributed by users on the virtual platforms [3].

Users’ knowledge contribution is critical to online communities but also difficult to sustain [13]. A key challenge faced by OIC is how to continually stimulate users’ continuous innovative knowledge contribution (CIKC) [14], which has been a recurring and prominent issue in open innovation research and practice [3,15]. It is crucial for enterprises to maintain validity. Therefore, research on the CIKC of members in OIC is urgent and necessary. We were encouraged to explore the following questions:

**Q1.**  *What factors can positively motivate community users to maintain the continuity of contributing innovative knowledge?*

**Q2.**  *Does the users’ psychological factor play any key role?*

**Q3.**  *Is there any mediator or moderator to be considered?*

The existing research on the motivations of user knowledge contributions in OIC has explored multiple theoretical and practical perspectives. According to previous research, external motivation can change users’ perceived benefits of knowledge contribution, which is drawn from the social exchange theory, thus enabling knowledge carriers to actively share knowledge, and boost knowledge diffusion, within an OIC, which can enhance the innovation performance of the community [16,17]. Moreover, researchers who studied the social network theory found that veteran members of OIC with higher intents on reputations usually have a better understanding of others’ needs and higher online communication skills, which can improve the constancy and effectiveness of knowledge contribution by reducing knowledge compilation costs and enhancing the accuracy of knowledge transfer within the community [18,19].

However, several research gaps in the literature remain prominent: 

Firstly, prior researchers of motivations in the open community mostly focused on the participation behaviors [7,10,20] while overlooking the aspect of knowledge management, which could also well explain the excitation mechanism in the sustainable development of OIC [3,21]. In this paper, we focused on innovation performance in the view of knowledge management.

Secondly, previous studies mainly focused on the quantity of knowledge contribution [13], overlooking the quality aspect. A prominent issue of OIC is the demand for users to generate high-quality ideas, which is crucial because a high quantity of engagement may include unnecessary, insufficient, or low-quality inputs, which may not necessarily bring quality to the creative library [22]. This study considers quantity and quality as two important but different aspects of CIKC since the significant differences in the quality of users’ knowledge contributions pose significant challenges to effectively meeting the needs of knowledge seekers and promoting innovation output [22,23].

Thirdly, it has been proven that users’ knowledge-contributing intentions or behaviors could be motivated by intrinsic cognitive factors, such as intrinsic enjoyment to help [24]. However, they lack a specific exploration of the external motivations based on social theories. The existing open innovation research lacks the identification of the interaction characteristics from a social perspective [25]. In addition, previous studies had insufficient explanations of the path analysis from the external environment to internal cognition, and then to knowledge contribution in OICs.

Thus, the theoretical and practical challenges mentioned above encouraged us to model a stimulus–organism–response framework to study the formation mechanisms of continuous knowledge contribution in OIC. In this paper, we focused on the factors of social benefits, along with users’ inner reactions, to study their important roles in the sustainable development of OIC, and discussed their interactions in the stimulation process. We have found that environmental stimulus (such as community relations and participation value) could have positive effects on the users’ continuous knowledge contribution, from both quantity and quality aspects, by means of stimulation onto inner response (self-efficacy). Moreover, the underlying mechanisms should also be considered along with personal factors, such as the knowledge level which showed a moderation role. The model was empirically validated with a survey of an open innovation community with a large volume of activities. And, we proposed effective incentive measures, including the utilization of user psychology, environmental stimulations, and users’ knowledge literacy, to improve CIKC.

## 2. Literature Review and Hypothesized Framework

### 2.1. Innovative Knowledge Contribution

#### 2.1.1. Open Innovation Community and Innovative Knowledge Contribution

In the age of big data, it is required for enterprises to introduce external innovative knowledge and resources across organizational boundaries rather than only internal resources to meet the needs of innovation [1,26]. The open innovation community is a popular type of virtual community, designed by enterprises as an important channel for enterprises to acquire external knowledge and information based on the internet [6,27]. The purpose of OIC construction is to meet the needs of enterprises to collect internal and external innovation resources and establish a community to carry out innovative activities and promote the dissemination of innovative knowledge [7,28]. OICs support user participation in knowledge sharing, which is inseparable from the improvement of users’ ideas [25]. In this study, we believed innovative knowledge contribution to be the interactions among the users to exchange knowledge and expertise to produce ideas and solutions, and to enhance affective and cognitive experiences by fostering collective intelligence. Cooperation in the form of posts and comments amongst online users is a critical component of the success of an internet-based community [11].

Knowledge contributors who have a better understanding of the needs of others and higher online information exchange skills [29] can improve the effect of knowledge diffusion within the community [30] by reducing the cost of knowledge compilation, and improving the accuracy of knowledge transfer within the OIC [31]. Meanwhile, it is not easy to encourage users to continuously contribute their knowledge, as many users might become inert after an initial surge of interest [32]. Thus, as for the OICs, providing stimuli to retain these community members to continuously contribute knowledge is helpful to improve innovations.

Considering online knowledge management, there are two main incentive measures: tool value and information value [33,34,35]. Tool value refers to the value gained from completing specific tasks, such as gaining reputation, solving problems, generating ideas, influencing others, and making decisions, by means of online social interactions effectively [36]. Information value refers to the value that individuals gain from sharing knowledge, acquiring knowledge, and understanding the thoughts of others [37]. In the OICs, these two motivations are commonly seen as community relations and participation value. 

#### 2.1.2. Community Relations and Innovative Knowledge Contribution

According to the social network theory, individual behavior is deeply influenced by others in social networks [3,33]. Based on this theory, scholars are convinced that there is a kind of social pressure which can lead to the change in individual behavior patterns, and increase the willingness to participate in innovation [33,34]. This social pressure grows accordingly with the increasingly significant socialization attributes in the virtual community [36]. Many scholars have pointed out that social relations, such as social connection, individual connection, and maintaining friendship, are important drivers of community participation [7,38].

Community relations refers to the connections formed by social interactions between users in open innovation communities [9,39]. CR is an important channel for knowledge contribution and information dissemination [22,40]. Support and encouragement can make community members feel more connected to the group, and make them more likely to remain devoted over time [22]. When it comes to OIC, knowledge and relation are two of the most common exchangeable products, whereas relation has become a rare commodity [41]. In an OIC, users can be followed by others. Users in OIC may not only seek and share knowledge, but also engage in social activities with individuals who share similar interests [10,42]. When the OIC establishes a strong community relationship among users, they will regard each other as friends, and will be willing to contribute ones’ knowledge when others encounter problems, which will positively promote users’ contribution behavior [31,43]. It is believed that peer recognition is effective in boosting users to higher states of contribution [12]. Studies have shown that those who hope to be accepted have a significant willingness to make social behaviors after being motivated by CR [44]. Moreover, people contribute their knowledge in order to earn respect and improve their reputation [45]. Studies have found that team members who perform well can gain a high degree of recognition, and promoted status, which in turn push them to work harder to contribute [46]. In other words, the more friends and followers in the community, the stronger the sense of responsibility and the need to provide more responsible knowledge and information. Moreover, in the process of contributing knowledge, users will pay attention to both the quantity and quality of contributions to receive others’ recognition [20]. Conversely, poor quality of the contributed knowledge will lead to the loss of interpersonal relationships and the decline of centrality in the community [20,47]. Therefore, this paper makes the following assumptions:

**Hypothesis** **1a (H1a).** *There is a significantly positive relationship between CR and the quantity of users’ CIKC in OIC.*

**Hypothesis** **1b (H1b).** *There is a significantly positive relationship between CR and the quality of users’ CIKC in OIC.*

#### 2.1.3. Participation Value and Innovative Knowledge Contribution

The social exchange theory points out that people carry out actions under the consideration of the possibility of obtaining benefits [26,48]. Knowledge sharing is a social interaction emphasized by the social exchange theory [37]. Participation value can be seen as a kind of direct benefit assessed by users in OIC [49]. When users measure the value of participating in OIC activities meaningfully and the knowledge environment of the community in line with expectations, they will spend more time and effort on knowledge contribution [50,51]. Social benefits and recognition are important drivers of voluntary contributions in online platforms which offer social interaction-oriented functionalities [52,53]. Higher value provided by a community can make its users experience a higher level of satisfaction, thus enhancing users’ loyalty, which is an important driving force [54]. So, the value of participation in OIC may increase users’ incentives to contribute their knowledge.

Only when users believe an OIC to be valuable would they participate in it [53,55]. Users who want to obtain higher PV would choose to engage the activities in an OIC which can provide a higher opportunity to meet their inner needs. Moreover, they would pay more attention to improving the quality, rather than the quantity, of knowledge when devoting contributions to help others and to meet the standard of a good knowledge environment in the community [56,57]. Therefore, we make the following assumptions:

**Hypothesis** **2a (H2a).** *The higher the PV, the greater the quantity of users’ CIKC in OIC.*

**Hypothesis** **2b (H2b).** *The higher the PV, the higher the quality of users’ CIKC in OIC.*

### 2.2. SOR Theoretical Framework

The stimulus–organism–response (SOR) theory, which originated in behavioral psychology, is a learning theory based on cognitivism [58]. The SOR model was first proposed by Mehrabian and Russell (1974) [59]. It consists of three parts: S stands for the stimulation stage, O stands for the psychological characteristics of individuals, and R stands for the individual reaction [59]. The SOR model is characterized by stimulating individual cognition through the integration of individual responses, thus eliciting a series of behaviors [58]. It explains how different environmental factors act as stimuli (S) that impact an individual’s internal state (O), leading to behavioral responses (R) [60]. Researchers often use the SOR theory, which has good and widespread applicability, to analyze user behavior in the context of social media interaction, online communities, online education, e-commerce, and other contexts [61].

Based on the SOR theoretical framework, this article took community relations and participation value as two dimensions of stimulus (S) according to the social network theory and social exchange theory, and viewed the process of “community relations and participation value—continuously knowledge contribution” as a stimulus–response (S-R) relationship.

According to Mehrabian and Russell (1974) [59], there should be a recognition element in the S-R relationship, and we believe that self-efficacy could make up the O part.

### 2.3. Mediation Role of Self-Efficacy

Self-efficacy refers to the internal cognition experienced by an individual on whether they can complete a certain work [62]. It was first proposed by Bandura in 1977, who believed that the level of self-efficacy directly determines the motivation level of individuals when they engage in certain activities [63].

It is found that people with higher confidence in expertise, skills, and abilities are more likely to provide useful content in online forums [3]. There is a strong correlation between their personal self-efficacy in providing knowledge and the amount of sharing they make [64]. When active users receive a lot of upvotes and followers for their contribution to the community, their reputation and self-efficacy rise [65]. It is claimed that users who believe the community is valuable may be satisfied with the community and may become loyal members, which encourages them to respond to the community by providing trustworthy and authentic information [66]. Knowledge contributions in OIC are mostly encouraged by positive feedbacks, which will increase the knowledge contributor’s feeling of self-efficacy, competence, and responsibility, and encourage them to contribute even more in the future [67]. Therefore, we make the following assumptions:

**Hypothesis** **3a (H3a).** *SE plays a mediator role between CR and the quantity of CIKC.*

**Hypothesis** **3b (H3b).** *SE plays a mediator role between CR and the quality of CIKC.*

**Hypothesis** **4a (H4a).** *SE plays a mediator role between PV and the quantity of CIKC.*

**Hypothesis** **4b (H4b).** *SE plays a mediator role between PV and the quality of CIKC.*

In sum, the stimulus that can trigger user behavior and response in the community ultimately affects the continuous innovative knowledge contribution (quantity and quality) of users through influencing user self-efficacy in open innovation communities. Thus, we constructed a SOR model in which community relations and participation value were seen as stimuli, and self-efficacy as recognition by the organism, and continuous innovative knowledge contribution as response. Although there was a lot of research on the factors of the knowledge contribution in virtual communities, few of them had studied the mediation mechanism of the self-efficacy of users in an OIC, and we intended to fill this blank in research.

### 2.4. Moderation Role of Knowledge Level

The quantity and quality of knowledge held by the members of virtual communities are uneven, i.e., there are differences in the breadth and depth of knowledge distribution [15], which is recognized as the difference in knowledge level [68]. Some members have cutting-edge, important, comprehensive, and high-end professional knowledge, while others have only general, highly formatted knowledge [69]. This difference in KL is the main cause of knowledge flow [70]. The higher the level of knowledge, the more likely it is for an individual to disseminate knowledge and be able to actively answer questions raised by the other members of the community [71]. Conversely, those who have a lower knowledge level are more likely to absorb knowledge [72]. It is because when community members perceive a large gap in knowledge level with others, they will choose to hide and make no knowledge contribution in order to avoid the risk of making mistakes [71]. The self-awareness of a high knowledge level, usually along with the self-efficacy of being capable of providing useful knowledge, will encourage users’ willingness to contribute [24,73].

In OIC, by answering questions and posting articles, individuals can share their knowledge to gain a sense of satisfaction for assisting others and potential returns [69]. Moreover, the higher the level of knowledge, the higher the quality of knowledge output [73]. And with higher knowledge advantages, the users of high-level knowledge will be more willing and encouraged to contribute knowledge, resulting in a more continuous knowledge contribution behavior [74]. Therefore, this paper makes the following assumptions:

**Hypothesis** **5a (H5a).** *KL can positively moderate the effect of CR on the quantity of CIKC.*

**Hypothesis** **5b (H5b).** *KL can positively moderate the effect of CR on the quality of CIKC.*

**Hypothesis** **6a (H6a).** *KL can positively moderate the effect of PV on the quantity of CIKC.*

**Hypothesis** **6b (H6b).** *KL can positively moderate the effect of PV on the quality of CIKC.*

Although former researchers had studied the impact of knowledge level on the knowledge contribution in virtual communities [27], they had not studied the moderation role of it. Thus, we intended to fill the gap in the moderation mechanism of knowledge level.

**Figure 1:**
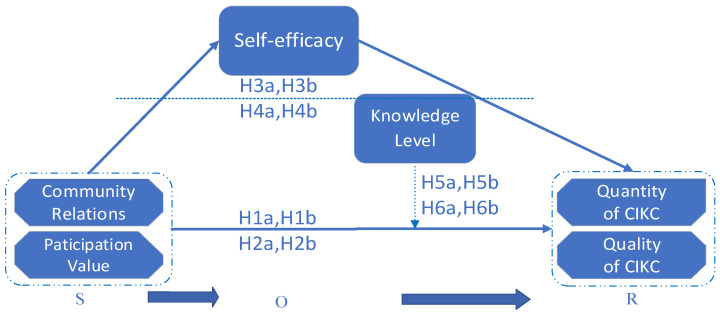
The theoretical research model.

To sum up, this study introduced the moderating variable—knowledge level—and explored how user’s SE in open innovation communities affects sustained knowledge contribution (from both quantity and quality aspects) under the influence of knowledge level. Therefore, this article combines the social network theory, social exchange theory, and SOR theory to construct a conceptual model (see Figure 1) to investigate the influence of CR and PV, and the mediation role of SE, and the moderation role of knowledge level.

## 3. Materials and Methods

### 3.1. Measurement and Data Collection

Data for this study came from the Xiaomi community (https://www.xiaomi.cn (accessed on 1 January 2022)), which is one of the most popular open innovation communities in China. The products and customers of Xiaomi are spread all over the world, and the Xiaomi community is the official application of Xiaomi Company, Beijing, China. By of the end of 2022, its registered users have exceeded 120 million, covering more than 200 countries and regions worldwide. The Xiaomi community is divided into different sections and regularly holds interactive activities, online live broadcasts, technical exchanges, and other activities through various means. Every day, many users post content, reply to messages, and participate in activities on social media platforms, presenting a very high level of activity and interactivity. This study used the Python 3.6 to collect user data from the three most active sections of Xiaomi community (Xiaomi community, MIUI Comprehensive Discussion, and Xiaomi Paper OS) during the one-year period of 2022. Considering the time attribute of user’s data, the observation period should be based on time periods, so we gathered the data for 6 periods in one year with every 2 months as a period.

Banerjee et al. (2023) considered users’ posts in virtual communities as the basic form of their participation in knowledge contribution [66]. Therefore, this article measures the knowledge contribution of users by tracking their posts over a period. The Xiaomi community provides a feedback mechanism through which each user can express their recognition and appreciation for the quality of the knowledge content when they browse. Therefore, this article selects the positive feedback received by users, namely the number of likes, as an indicator to measure the quality of knowledge contribution. The most direct expression of trust is “adding followers”, so this study chose the number of followers in the community to measure the strength of user community relationships. It was convinced that users measure the knowledge environment of the community to determine whether the community and the tasks it publishes have participation value [60]. The Xiaomi community consists of about one hundred forums, belonging to various aspects such as different products, systems, comprehensive discussions, applications, smart living, etc. When users believe that the forum and its published tasks have participatory value, they will join the community forums. Therefore, this article considers the number of users’ forums as the value of participation. The Xiaomi community offers users different types of medals, including the behavior medal, memorial medal, honor medal, etc., to encourage their sense of honor in the open innovation community. When users receive medals from the community, it increases their sense of satisfaction and recognition, making them believe that they can complete more community tasks, thereby enhancing their self-efficacy. Moreover, the difficulty level of obtaining different honor medals varies, and the medal honor value is calculated based on all the medals obtained, combined with the difficulty coefficient of obtaining each medal. Therefore, medal honor values can be used to calculate users’ self-efficacy in the community. In the Xiaomi community, the system divides users into 10 different user groups (levels from 1 to 10) based on their knowledge and professional level in the community. Therefore, this study measures users’ knowledge levels using their group levels (from 1 to 10).

In summary, apart from user personal information, our target data includes the number of user’s posts (representing the quantity of CIKC), number of likes received by user (representing the quality of CIKC), sum of user’s followers (representing CR), number of user’s forums participated in (representing PV), user’s value of medal honor (representing SE), and user’s level in OIC (representing KL). Table 1 summarizes the variables and their descriptions employed in our study.

### 3.2. Data Pre-Analysis and Model Specification

Since only the data of the users whose observation data differ in a continuous period will be valuable and explanatory, those users who have no difference in all the observed data items in the six consecutive observation periods were eliminated, and finally, a dataset of 18,410 active users was obtained. We standardized these variables before analysis to eliminate the dimensional relationships between the variables and to render the data comparable. Table 2 provides the summary statistics and correlation matrix for the main variables of the dataset.

Firstly, it is necessary to check the multiple collinearities of the model. Based on a correlation analysis, the correlation coefficients between the variables were all less than 0.5. This result basically leads to the refusal of the collinearity assumption. Moreover, the statistical basic situation and variance inflation factor (VIF) of all the variables were analyzed based on an OLS model. The model estimation would be distorted when there was a high correlation between the explanatory variables, which means a multiple collinearity problem. Calculating the VIF value in the regression analysis can be used to exclude multicollinearity problems [38]. In the study, the results of the VIF of each variable were all far less than 5, so we were convinced that there was no multicollinearity between those dependent variables.

Secondly, SPSSPro was used to select the method of panel data model form. The comprehensive results of the F test, Breusch–Pagan test, and Hausman’s test supported the choice of the fixed effect model. In addition, considering the bias of model estimation that individual and time differences could cause, we used the wwo-way fixed effect model with both individual fixed effects and time-fixed effects to estimate the effect of CR and PV on the quantity and quality of CIKC.

Baron and Kenny (1986) were convinced that the mediation construct is intermediate between the other two constructs [75]. Changes in the endogenous structure will reflect exogenous changes in the structure caused by the differences in the mediation structure [76]. Hierarchical multiple regression is commonly used in research on mediating or moderating effects. Therefore, in this paper, CR and PV were regarded as the explanatory variable X, SE as the mediator variable M, KL as the moderator, and quantity and quality of CIKC as the interpreted variable Y. To study the relationships among these variables, the following mediation effect model and the moderation effect model were established by referring to the method of Baron and Kenny (1986) [75]:

Model I (mediation effect model, *M_it_* = self-efficacy):

(1) $$Y_{it}=c_{0}+cX_{it}+\mu_{i}^{1}+\varphi_{t}^{1}+\varepsilon_{i,t}^{1}$$

(2) $$M_{it}=a_{0}+aX_{it}+\mu_{i}^{2}+\varphi_{t}^{2}+\varepsilon_{i,t}^{2}$$

(3) $$Y_{it}=c_{0}^{\prime }+c^{\prime }X_{it}+bM_{it}+\mu_{i}^{3}+\varphi_{t}^{3}+\varepsilon_{i,t}^{3}$$

Model II (moderation effect model, *U_it_* = knowledge level):

(4) $$Y_{it}=c_{0}+c_{1}X_{it}+c_{2}U_{it}+\mu_{i}^{1}+\varphi_{t}^{1}+\varepsilon_{i,t}^{1}$$

(5) $$Y_{it}=a_{0}+a_{1}X_{it}+a_{2}U_{it}+a_{3}U_{it}X_{it}+\mu_{i}^{2}+\varphi_{t}^{2}+\varepsilon_{i,t}^{2}$$

In the equations, $Y_{\mathrm{it}}=\left\{\begin{array}{c} Quantity_{\mathrm{it}}\\ Quality_{\mathrm{it}} \end{array}\right\}$ represent the quantity and quality of continuous knowledge contribution, $X_{\mathrm{it}}=\left\{\begin{array}{c} {CR}_{\mathrm{it}}\\ {PV}_{\mathrm{it}} \end{array}\right\}$ represent the community relations and participation value of users, and a, b, c, $c^{\prime }$, $a_{i}$, and $c_{i}$ represent the coefficients to be estimated. And $\mu_{i}^{k}$ captures the unobserved user-specific effects, $\varphi_{t}^{k}$ controls for the time-fixed effects, and $\varepsilon_{i,t}^{k}$ is the error term. The models enable us to make full use of the characteristics of our data to estimate the models and test our hypotheses efficiently.

## 4. Results

### 4.1. Main Effect Test and Test of Mediation Effect

The results of the main effects are reported in Table 3, which presents the estimated coefficients, the associated *p*-values, and the overall explanatory power of the models.

Firstly, this paper examined the mediating effect of SE between the independent variables of CR and PV and the dependent variable of the quantity and quality of CIKC through three-stage regression. The mediation effect test regression results are shown in the table. The coefficient of c in model 1 is 0.227, which is significantly positive at the level of 0.001, indicating that CR positively affects the quantity of CIKC in OIC, so H1a is supported. The same conclusions go to the hypotheses of H1b, H2a, and H2b, as the coefficients of c in the models 2,3 and 4 are 0.372, 0.293, and 0.377, respectively, and all of them are significant at the level of 0.001. Together with the other coefficients of a = 0.464, b = 0.258, and c′ = 0.108, all were significant at the level of 0.001, in model 1, with the value of z (Sobel test) = 2.296 with *p* = 0.022 < 0.05 indicating that it had passed the Sobel test, and we can draw the conclusion that there is a partial mediation effect of SE between CR and the quantity of CIKC, which means H3a is supported.

In addition, according to Hair et al. (2014) [77], researchers could calculate the strength of intermediaries by means of the ratio of the indirect effects to total effects, which is also called the variance accounted for (VAF) value. Moreover, it is believed that there is a full mediation effect when VAF > 80%, a partial mediation effect if 20% < VAF < 80%, and no mediation effect if VAF < 20% (Hair et al., 2014) [77]. So, the magnitude of the mediation effect was also calculated in model 1, and the calculation of VAF, namely $\frac{ab}{ab+c^{\prime }}$ was 52.57%, larger than 20%, indicating that there was a partial mediation effect. The same conclusions go to hypothesis H4a, i.e., there was a partial mediation effect of SE on the process from PV to the quantity of CIKC. Nevertheless, the hypotheses of H3b and H4b were not supported, because the VAF of $\frac{ab}{ab+c^{\prime }}$ = 10.11% in model 2 was lower than 20%, and the significance of the coefficient of b in model 4 was not significant.

### 4.2. Moderation Effect Test

**Figure 2:**
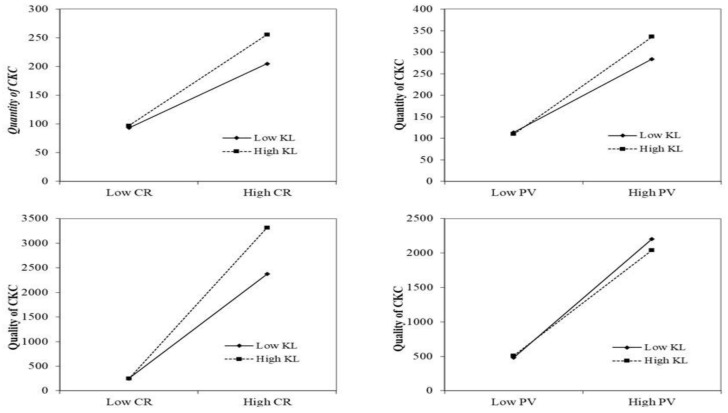
Moderation Effect Test.

In this paper, the moderation effects of knowledge level on the process of “CR or PV to the quantity or quality of CIKC” was tested by four-stage panel-data fixed effect regression, and the test results are also shown in Table 3. The moderation effect could be confirmed only when the coefficients of $c_{1}$, $a_{1}$, and $a_{3}$ in model II were all significant. As shown in the table, we can draw the conclusion that H5a was supported, since $c_{1}$ (=0.227), $a_{1}$ (=0.167), and $a_{3}$ (=0.076) were all significant at the level of 0.001. The same conclusions go to the hypotheses of H5b and H6a. In other words, KL can positively moderate the effects of CR on the quantity of CIKC, CR to the quality of CIKC, and PV to the quantity of CIKC. Figure 2 depicts the moderation effects of KL.

There is an interesting finding that the coefficients of $c_{1}$ (=0.377), $a_{1}$ (=0.411), and $a_{3}$ (=−0.041) related to hypothesis H6b were all significant at the level of 0.01, but the coefficient of $a_{3}$ was a negative value. It means that although hypothesis H6b was not supported, it had a reversed conclusion that KL can negatively moderate the effect of PV on the quality of CIKC.

### 4.3. Robustness Checks

It was necessary to test the robustness of the fixed effect model (Hair et al., 2014) [77]. This article uses the surrogate variable method to test the robustness of the model by replacing the intermediary variable in the model and testing the mediating effect of SE on the impact of incentive factors on sustained knowledge contribution.

When we chose the value of the medal honor of users as the mediation variable, we also found the number of honored medals could be like the value of medal honor as a representation of SE. Therefore, this article selected the number of honored medals to measure the SE level. Poisson regression analysis was conducted on the mediating effects and the results are shown in Table 4. Based on the results, we can draw the same conclusion of the mediation effects as above.

## 5. Discussion

The continuous innovative knowledge contribution of users is of great significance to the sustainable development of the open innovation community. This research analyzed the factors of the quantity and quality of users’ continuous innovative knowledge contributions.

The study verified the hypotheses that community relations positively affect the quantity and quality of users’ sustained knowledge contributions in open innovation communities. This conclusion is coordinated with that of Ozturk et al. (2023) [22], and the mediation hypothesis that community relations increase the quantity of sustained knowledge contributions by enhancing users’ self-efficacy was supported. In the community, users usually treat community relations like friendships, so they are more willing to contribute knowledge to help others when being asked questions, thus promoting continuous innovative knowledge contributions. Moreover, the strength of community interpersonal relationships is also the embodiment of the user’s status in the community, which can encourage the user’s self-belief. This sense of support and belonging helps establish ongoing relationships between users, which can involve sharing information and providing feedback. Since the scope of knowledge dissemination will expand with the enhancement of community interpersonal relations, users will continue to contribute knowledge, participate in community activities, and strengthen communication and exchanges with other users in order to maintain their community interpersonal relationships and community status. Users’ perception of their abilities and willingness to share their ideas increase when they know their contributions receive attention from other individuals. Therefore, community relations positively influence the quantity and quality of users’ ongoing knowledge contributions in open innovation communities, and self-efficacy plays a mediation role.

The hypotheses that participation value positively affects the quantity and quality of users’ sustained knowledge contributions in the open innovation community were supported. When users perceive a good knowledge environment of an open innovation community, they will contribute highly qualified knowledge consistent with the environment, and the quality of continuous innovative knowledge contribution will be improved. Conversely, a lack of value recognition creates uncertainty in user participation and reduces user contribution behavior, and a high degree of non-participation would be destructive. This conclusion seems consistent with that of Wang et al. (2024) [25]. And, the mediation hypothesis that participation value increases the quantity of sustained knowledge contributions by enhancing self-efficacy was also supported. If users find the time and energy spent in the OIC meaningful, they will actively participate in the activities and continuously contribute knowledge. When meeting valuable tasks or problems, users who have the confidence to complete tasks are more likely to contribute their knowledge. Therefore, the participation value positively affects the quantity and quality of users’ ongoing knowledge contributions in the open innovation community, and self-efficacy plays a mediation role.

However, users with high self-efficacy might not improve the quality of content when continuously contributing knowledge. This phenomenon might be explained by the fact that in social networks, the high effectiveness of users’ innovative idea contribution requires not only strong mutual influence from other members of the community but also the avoidance of exposure to too much similar ideas. Because, the redundancy of connections might cause analogical transfer from past experiences and other users’ ideas, and finally to a cognitive fixation, which usually appears to have a prominently negative impact on innovations. On the other hand, although higher users’ self-efficacy usually combines with higher enthusiasm to contribute innovative ideas, users with low knowledge levels may not necessarily complete high-level tasks well, even if they have the confidence to do so. They should rather make more efforts to increase their knowledge level to improve their quality of knowledge contribution than simply focus on ordinary participation activities.

In this article, knowledge level refers to the difference in knowledge available among community users. The hypotheses fully supported that with higher KL, the effect of community relations seems to be stronger to increase both the quantity and quality of sustained knowledge contributions. The higher the user’s knowledge level, the greater their knowledge reserve, and the higher their specialization level. Specialization positively affects both idea generation and implementation. It is much easier for higher knowledge-level users to participate in the discussions, then the quantity of knowledge contributions will increase accordingly. As an indirect reward provided by the community, CR will increase the users’ knowledge contribution in the open innovation community to maintain their community status, which is in turn representing specialization.

The hypothesis that the higher the level of knowledge, the stronger the effect of participation value to improve the quantity of sustained knowledge contribution was supported. After measuring the knowledge environment of the open innovation community, users will use their strong knowledge reserves to contribute knowledge in order to propose knowledge that conforms to the knowledge environment of the open innovation community when they encounter tasks that can be solved when participating in open innovation community activities, leading to the increase in the quantity of CIKC.

However, the hypothesis that the higher the level of knowledge, the stronger the effect of participation value in increasing the quality of sustained knowledge contributions is not valid, and even reversed. This is a novel conclusion, and this phenomenon might be explained by the fact that higher knowledge-level users tend to make more refined efforts on high-level engagement rather than focusing on daily affairs. On the contrary, the inferior quality of low knowledge-level member participation might negatively impact the community atmosphere and outcomes, and become a fundamental problem for innovation communities’ viability. The higher-level members should be allowed to contribute less engagement in low-level participation in order to save efforts to mainly focus on more important targets instead of being distracted from the community’s original intention of innovation.

This study firstly promotes the application of the social network theory and social exchange theory in open innovation research, and these findings mentioned above help us to strengthen the extant relevant consensus of external incentives to promote innovative behavior and innovation performance. Moreover, this paper fills in the gap in how inner psychological factors play a role. It contributes by extending the application of self-efficacy in online innovation communities and examining the impact of mediation. Thirdly, this study creatively introduces knowledge level as a moderating variable. The results reveal that knowledge plays different moderating roles between incentives and the quantity and quality of CIKC. This study provides insights into how external incentives interact with inner self-efficiency and then influence on continuous innovative knowledge contributions. It also provides several managerial implications for online innovation community management.

This paper was an exploration attempt at the factors of the continuous innovative knowledge contribution of users in OIC. Yet, it was difficult to ensure the comprehensiveness of consideration and there were still some shortcomings, which can be further studied. To be specific, when examining the influence factors, more stimuli should be considered to make a more comprehensive picture. Moreover, although its typicality has been discussed, the sample selection of the Xiaomi community as the only platform might have lost the universality of empirical research conclusions, and it might need to be further tested on more platforms. Lastly, since it was difficult to obtain the replacement variables of moderation, the surrogate variable method to test the robustness of the moderation model seemed a little unconvincing. In future research, we should consider selecting alternative online interactive communities and studying more replacement variables to validate our conclusions.

## 6. Conclusions

This research focused on the influence mechanism of community relations, participation value, self-efficacy, and knowledge level on the continuous innovative knowledge contribution of members in the open innovation community. Specifically, this paper selected an active innovation community to conduct research and summarized the motivators and incentive mechanisms of user knowledge contribution based on the empirical analysis results.

The results of this field study show that both community relations and participation value will positively affect the quantity and quality of users’ sustained knowledge contributions in open innovation communities. Self-efficacy plays a mediation role between these two incentives and the quantity, rather than the quality, of users’ sustained knowledge contributions. Knowledge level moderates the relationships between these two incentives to users’ sustained knowledge contributions. However, there is a reversed moderation effect of knowledge level on the process from participation value to the quality of users’ sustained knowledge contributions.

This study has very important theoretical implications. We integrated the social network theory and social exchange theory to explore the factors influencing the continuous innovative knowledge contribution of users in the open innovation community and their influencing mechanisms, and carried out user-centered research on the quantity and quality of continuous innovative knowledge contribution. Moreover, the empirical analyses were carried out by the panel-data fixed effect regression test, and the three-stage and four-stage hierarchical regression methods.

This study has also very important practical implications. We proposed effective incentive measures in order to continuously improve users’ self-efficacy, stimulate and maintain community vitality, and rebuild a more complete sustainable community knowledge supply mechanism based on maintaining community activities.

As a kind of virtual community, the social open innovation community has made great changes in people’s knowledge contribution and communication methods, and the prosperity of the community has also brought great benefits to the community itself and the users and groups that establish personal brands in the community, and more and more communities are rising and developing. Retaining community members to continuously participate in virtual community platform activities and contribute knowledge is one of the core tasks of open innovation community operation managers. The research on the influencing factors of users’ continuous innovative knowledge contribution will not only help the open innovation community retain the existing users, but also help attract potential new users, and improve the activity and attractiveness of the community. It will help open innovation community managers to quickly and efficiently meet the needs of users, promote further growth of the community, and obtain a higher innovative knowledge repository.

This study advocates leveraging OICs as a means of informal knowledge management. Community management should emphasize the impact of community relations and participation values on continuous knowledge contribution. For the open innovation community platform, it needs to actively improve the reward mechanism, and meanwhile, take the users’ inner reactions into consideration, in order to stimulate users to continually contribute knowledge. Community managers should take effective measures to maintain an innovative environment for higher knowledge-level users to continuously contribute knowledge. Managers should make efforts to encourage the community users to improve their knowledge level to increase their quality of knowledge contribution.

As for the open innovation community users, it is important to take the initiative to establish community relationships with other community members. By maintaining a good relationship with other users, they can not only solve their own problems but also improve satisfaction and the sense of achievement due to the improvement in the community status. It is also necessary to continuously expand their knowledge base. Only by expanding their knowledge reserves and improving their knowledge level would users be able to properly solve problems and output high-quality knowledge, thereby gaining community rewards, reputations, and respect.

## Figures and Tables

**Table 1:** Variables and their descriptions.

Variable Type	Variable	Metric	Description
Dependent Variable	Quantity of CIKC	Quantity_it_	Number of posts by user i in period t
	Quality of CIKC	Quality_it_	Number of likes received by user i in period t
Independent Variable	Community Rations	CR_it_	Number of user i’s followers in period t
	Participation Value	PV_it_	Number of user i’s forums in period t
Mediator	Self-efficacy	SE_it_	Value of medal honor of user i in period t
Moderator	Knowledge Level	KL_it_	The level of user i in period t

**Table 2:** Descriptive summary of variables.

	Variables	Mean	SD	1	2	3	4	5	6	VIF
1	Quantity_it_	34.46	290.574	1						
2	Quality_it_	23.30	333.442	0.245 **	1					
3	CR_it_	19.71	27.439	0.227 **	0.372 **	1				1.698
4	PV_it_	21.10	268.832	0.448 **	0.377 **	0.635 **	1			2.154
5	SE_it_	41.36	846.769	0.308 **	0.236 **	0.464 **	0.617 **	1		1.639
6	KL_it_	6.00	37.717	0.006 (n.s.)	0.002 (n.s.)	0.002 (n.s.)	0.008 (n.s.)	0.006 (n.s.)	1	1.000

Notes. ** *p* < 0.001; ‘n.s.’ = not significant.

**Table 3:** Results of the structural model.

Models		X = Community Relations		X = Participation Value	
		Coefficient β (*p*-Value)	Decision	Coefficient β (*p*-Value)	Decision
**Model I**	**Y = quantity of CIKC**	Model 1:		Model 3:	
		c = 0.227 (0.000 **)	H1a: Supported	c = 0.448 (0.000 **)	H2a: Supported
		a = 0.464 (0.000 **)		a = 0.617 (0.000 **)	
		b = 0.258 (0.000 **)		b = 0.250 (0.000 **)	
		c′ = 0.108 (0.000 **)	H3a: Supported	c′ = 0.294 (0.000 **)	H4a: Supported
		z (Sobel test) = 2.296 (*p* = 0.022)		z (Sobel test) = 3.394 (*p* = 0.0007)	
		$\frac{ab}{ab+c^{\prime }}$ = 52.57%		$\frac{ab}{ab+c^{\prime }}$ = 34.43%	
	**Y = quality of CIKC**	Model 2:		Model 4:	
		c = 0.372 (0.000 **)	H1b: Supported	c = 0.377 (0.000 **)	H2b: Supported
		a = 0.464 (0.000 **)		a = 0.617 (0.000 **)	
		b = 0.081 (0.000 **)		b = 0.005 (n.s.)	
		c′ = 0.334 (0.000 **)	H3b: Unsupported	c′ = 0.374 (0.000 **)	H4b: Unsupported
		$\frac{ab}{ab+c^{\prime }}$ = 10.11%		$\frac{ab}{ab+c^{\prime }}$ = 0.82%	
**Model II**	**Y = quantity of CIKC**	Model 5:	H5a: Supported	Model 7:	H6a: Supported
		$c_{1}$ = 0.227 (0.000 **)		$c_{1}$ = 0.448 (0.000 **)	
		$a_{1}$ = 0.167 (0.000 **)		$a_{1}$ = 0.402 (0.000 **)	
		$a_{3}$ = 0.076 (0.000 **)		$a_{3}$ = 0.057 (0.000 **)	
	**Y = quality of CIKC**	Model 6:	H5b: Supported	Model 8:	H6b: Reversed conclusion (negative moderation effect)
		$c_{1}$ = 0.372 (0.000 **)		$c_{1}$ = 0.377 (0.000 **)	
		$a_{1}$ = 0.275 (0.000 **)		$a_{1}$ = 0.411 (0.000 **)	
		$a_{3}$ = 0.121 (0.000 **)		$a_{3}$ = −0.041 (0.001 *)	

Notes. * *p* < 0.01; ** *p* < 0.001; ‘n.s.’ = not significant; ab = cross term of Sobel test.

**Table 4:** Results of the structural model (surrogate variable method).

Models		X = Community Relations		X = Participation Value	
		Coefficient β (*p*-Value)	Decision	Coefficient β (*p*-Value)	Decision
**Models I**	***Y = quantity of CIKC***	*Model 1:*		*Model 3:*	
		c = 0.227 (0.000 **)	*H1a: Supported*	c = 0.448 (0.000 **)	*H2a: Supported*
		a = 0.424 (0.000 **)		a = 0.583 (0.000 **)	
		b = 0.250 (0.000 **)		b = 0.221 (0.000 **)	
		c′ = 0.121 (0.000 **)	*H3a: Supported*	c′ = 0.319 (0.000 **)	*H4a: Supported*
		z (Sobel test) = 4.688 (*p* = 0.000)		z (Sobel test) = 6.727 (*p* = 0.000)	
		$\frac{ab}{ab+c^{\prime }}$ = 46.70%		$\frac{ab}{ab+c^{\prime }}$ = 28.76%	
	***Y = quality of CIKC***	*Model 2:*		*Model 4:*	
		c = 0.372 (0.000 **)	*H1b: Supported*	c = 0.377 (0.000 **)	*H2b: Supported*
		a = 0.424 (0.000 **)		a = 0.583 (0.000 **)	
		b = 0.091 (0.000 **)		b = 0.019 (n.s.)	
		c′ = 0.333 (0.000 **)	*H3b: Unsupported*	c′ = 0.367 (0.000 **)	*H4b: Unsupported*
		$\frac{ab}{ab+c^{\prime }}$ = 10.37%		$\frac{ab}{ab+c^{\prime }}\;$ = 2.94%	

Notes. ** *p* < 0.001; ‘n.s.’ = not significant; ab = cross term of Sobel test.

## Data Availability

The data are not publicly available due to data privacy laws.
